# Marijuana and Myocardial Infarction in the UK Biobank Cohort

**DOI:** 10.7759/cureus.22054

**Published:** 2022-02-09

**Authors:** Steven Lehrer, Peter H Rheinstein

**Affiliations:** 1 Radiation Oncology, Icahn School of Medicine at Mount Sinai, New York City, USA; 2 Family Medicine, Severn Health Solutions, Severna Park, USA

**Keywords:** myocardial infarct, heart, risk factors, smoking, marijuana

## Abstract

Background: Atrial fibrillation, ventricular tachycardia, acute coronary syndromes, and cardiac arrest have been attributed to marijuana. But the National Academy of Science’s 2017 Report, *The Health Effects of Cannabis and Cannabinoids*, found limited evidence that acute marijuana smoking is positively associated with an increased risk of acute myocardial infarction, and uncovered no evidence to support or refute associations between any chronic effects of marijuana use and increased risk of myocardial infarct (MI).

Aims: We sought to determine the association of marijuana smoking with MI in the UK Biobank cohort. Because red wine is a mood-altering substance, we compared the effect of marijuana with red wine on MI incidence.

Methods: Our analysis included all subjects with MI. The diagnosis was ascertained using the 10^th^ Revision of the International Classification of Diseases (ICD10 I21). Marijuana was recorded in UKB Category 143, medical conditions, marijuana use. Cigarette smoking information was from UKB Category 100058, smoking. To compare marijuana smoking with the effect of wine drinking we used data from UKB Category 10051, alcohol.

Results: With marijuana use, MI incidence decreased (p < 0.001, two tail Fisher exact test). Red wine was associated with lower MI incidence, although the incidence begins to rise at 11 or more glasses per week (p < 0.001, two tail Fisher exact test). Multivariate analysis was done with logistic regression, MI dependent variable, cigarette pack-years, diabetes type 2, sex, BMI, hypertension, marijuana use, age, red wine consumption, independent variables. Odds ratio (O.R.) 0.844 associated with marijuana use indicates that MI was less likely in marijuana users and was comparable to the effect of red wine (O.R. 0.847).

Conclusion: Marijuana, which has not been shown to have the favorable physiologic effects of red wine on the heart, does reduce MI risk to an extent comparable to red wine. Perhaps both affect the heart by reducing stress.

## Introduction

DeFilippis et al. have reported increased use of marijuana over time among young adults who experienced a myocardial infarct (MI) [1]. Atrial fibrillation, ventricular tachycardia, acute coronary syndromes, and cardiac arrest have been attributed to marijuana. The subjects were young and had no significant cardiovascular risk factors [2]. Although the effect on coagulation is unclear, marijuana may have adverse cardiovascular effects at large doses [3].

The National Academy of Science’s 2017 Report, The Health Effects of Cannabis and Cannabinoids, found limited evidence that acute marijuana smoking is positively associated with an increased risk of MI, and uncovered no evidence to support or refute associations between any chronic effects of marijuana use and increased risk of MI [4,5].

The recent increase in marijuana smoking and the legalization of marijuana in multiple states have led to significant public health debate and the need to better understand the cardiovascular health effects. In the current analysis, we sought to determine the association of marijuana smoking with MI in the UK Biobank cohort. Because red wine is a mood-altering substance, we compared the effect of marijuana with red wine on MI incidence.

## Materials and methods

The UK Biobank is a large prospective observational study of men and women. Participants were recruited from across 22 centers located throughout England, Wales, and Scotland between 2006 and 2010 and continue to be longitudinally followed for the capture of subsequent health events [6]. Follow-up health information is provided by linkage to primary care electronic health records, death and cancer registries, and hospital admission records [7].

Inclusion criteria

Our UK Biobank application was approved as UKB project 57245 (S.L., P.H.R.). Our analysis included all subjects with MI. The diagnosis was ascertained using the 10th Revision of the International Classification of Diseases (ICD10 I21). Marijuana was recorded in UKB Category 143, medical conditions, marijuana use. Cigarette smoking information was from UKB Category 100058, smoking. To compare marijuana smoking with the effect of wine drinking we used data from UKB Category 10051, alcohol.

Exclusion criteria

We did not include subjects missing any of the inclusion criteria or make corrections for missing data.

The severity of MI, ST-elevation myocardial infarction (STEMI) or non-ST-elevation myocardial infarction (NSTEMI), are from the following UK Biobank data fields:

· 4002 - Date of STEMI, earliest reported STEMI for a participant

· 4003 - Source of STEMI report, noted in the Results section below

· 4004 - Date of NSTEMI, earliest reported NSTEMI for a participant

· 4005 - Source of NSTEMI report, noted in the Results section below

Data processing was performed on Minerva, a Linux mainframe with Centos 7.6, at the Icahn School of Medicine at Mount Sinai. We used the UK Biobank Data Parser (ukbb parser), a python-based package that allows easy interfacing with the large UK Biobank dataset [8]. SPSS v25 (IBM, Armonk, NY, USA) was used for data analysis.

## Results

**Figure 1:**
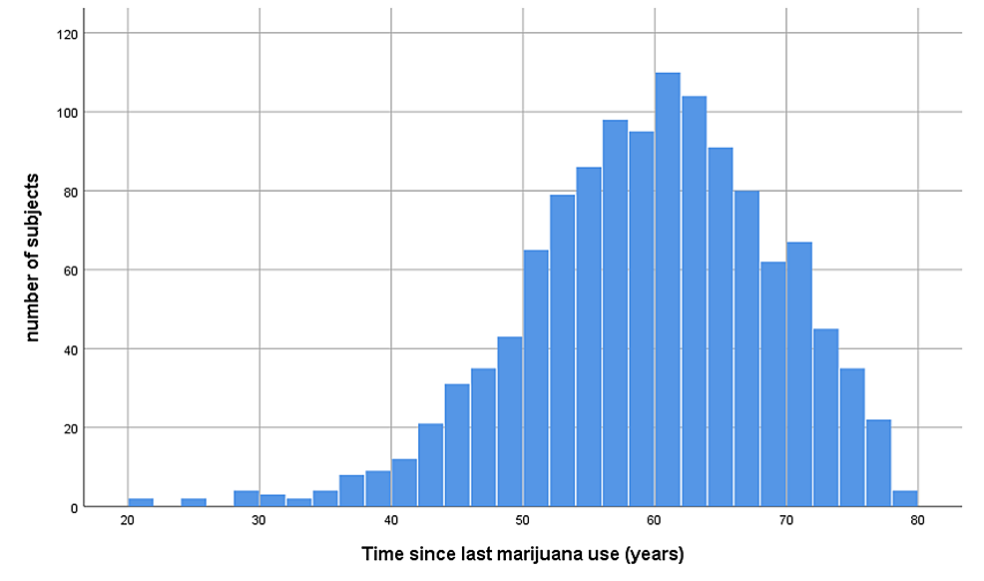
Time gap between last marijuana use and MI. MI - myocardial infarct

The demographics of the study population are given in Table 1. MI was identified by self-report in 25.4% of subjects, hospital admission data in 71.9% of subjects, death data only 2.7% of subjects. The time gap between last marijuana use and MI is shown in Figure 1.

**Table 1:** Demographic data for subjects in this study. MI - myocardial infarct

	MI	No MI
N	15,574	486,919
Sex	74% male	45% male
Age	60±6.8	56±8
Hypertension	15%	6%
BMI	28.7±4.7	27±4.7
Pack-years	31±22	23±19
Diabetes type 2	18%	5.6%

Marijuana use versus MI is given in Table 2. With marijuana use, MI incidence decreased (p < 0.001, two tail Fisher exact test).

**Table 2:** Marijuana use versus myocardial infarct. With increasing marijuana use, myocardial infarct incidence decreased (p < 0.001, two tail Fisher exact test).

		MI	Ml	
marijuana use		no	yes	Total
none		119680	2780	122460
	% within MI	77.90%	81.90%	77.90%
1-2 times	Count	14578	270	14848
	% within MI	9.50%	8.00%	9.50%
3-10 times	Count	8454	147	8601
	% within MI	5.50%	4.30%	5.50%
11-100 times	Count	6886	110	6996
	% within MI	4.50%	3.20%	4.50%
more than 100 times	Count	4119	87	4206
	% within MI	2.70%	2.60%	2.70%
Total	Count	153717	3394	157111
	% within MI	100.00%	100.00%	100.00%

Red wine consumption, glasses/week versus MI is given in Table 3. Red wine was associated with lower MI incidence, although the incidence begins to rise at 11 or more glasses per week (p < 0.001, two tail Fisher exact test).

**Table 3:** Red wine consumption, glasses/week versus MI. Red wine was associated with lower MI incidence, although the incidence begins to rise at 11 or more glasses per week (p < 0.001, two tail Fisher exact test). MI - myocardial infarct

Red wine		MI	MI	
Glasses/week		No	Yes	
0	Count	111,992	3,740	115,732
	% within MI	33.50%	36.90%	33.60%
1	Count	29,716	790	30,506
	% within MI	8.90%	7.80%	8.90%
2	Count	38,959	1,100	40,059
	% within MI	11.70%	10.90%	11.60%
3	Count	31,506	934	32,440
	% within MI	9.40%	9.20%	9.40%
4	Count	21,514	616	22,130
	% within MI	6.40%	6.10%	6.40%
5	Count	8,567	230	8,797
	% within MI	2.60%	2.30%	2.60%
6	Count	34,350	975	35,325
	% within MI	10.30%	9.60%	10.30%
7	Count	6,013	178	6,191
	% within MI	1.80%	1.80%	1.80%
8	Count	6,851	196	7,047
	% within MI	2.10%	1.90%	2.00%
9	Count	5,195	144	5,339
	% within MI	1.60%	1.40%	1.60%
10	Count	7,265	213	7,478
	% within MI	2.20%	2.10%	2.20%
11 or more	Count	32,259	1,013	33,272
	% within MI	9.70%	10.00%	9.70%

Logistic regression with 95% confidence intervals, lower bound (L.B.), upper bound (U.B.) is given in Table 4. MI is the dependent variable, cigarette packyears, diabetes type 2, sex, BMI, hypertension, marijuana use, age, red wine consumption, are independent variables. O.R. 3.152 associated with sex indicates that MI was more likely in men. O.R. 0.844 associated with marijuana indicates that MI was less likely in marijuana users and was comparable to the effect of red wine (O.R. 0.847, none versus 1-10 glasses per week).

**Table 4:** Logistic regression with 95% confidence intervals, lower bound (L.B.), and upper bound (U.B.). MI dependent variable, cigarette pack-years, diabetes type 2, sex, BMI, hypertension, marijuana use (yes or no), age, red wine consumption (none versus 1-10 glasses per week) independent variables. Odds ratio (O.R.) 3.152 associated with sex indicates that MI was more likely in men. O.R. 0.844 associated with marijuana indicates that MI was less likely in marijuana users and was comparable to the effect of red wine (O.R. 0.847).

	95% L.B.	O.R.	95% U.B.	P-value
Sex	2.647	3.152	3.753	<0.001
Age	1.034	1.046	1.058	<0.001
Marijuana	0.713	0.844	0.999	0.047
Hypertension	1.504	1.852	2.279	<0.001
Diabetes type 2	1.828	2.255	2.781	<0.001
Pack years	1.008	1.012	1.016	<0.001
BMI	1.002	1.018	1.034	0.029
Red wine	0.733	0.847	0.979	0.025

There was no correlation between marijuana use and severity of MI, STEMI, or NSTEMI (p = 0.6, chi-square test).

## Discussion

The association of marijuana use with reduced risk of MI is not entirely in accord with current assumptions about the cardiac effects of marijuana [5,9,10]. Yet marijuana cardioprotection may resemble that of red wine.

Moderate consumption of red wine helps to prevent coronary heart disease (CHD) through several mechanisms: increasing high-density lipoprotein cholesterol plasma levels, decreasing platelet aggregation, augmenting antioxidant effects, and restoring endothelial function [11]. Another mechanism could be a reduction of psychological stress, negative emotions, and resistance to social interactions especially evident in the so-called type D (distressed) personality, defined as a combination of negative affectivity (worry, irritation, melancholy) and social inhibition (reticence and a lack of self-assurance). Moderate marijuana consumption to reduce stress and induce a sense of well-being in type D personalities may be beneficial to the heart, like red wine, and diminish the risk of MI.

In the case of CHD, type D personality may have a negative impact on health. In individuals with chronic heart failure, type D personality is linked to a worsening health state and an increase in depressive symptoms. Following cardiac rehabilitation or coronary artery bypass grafting (CABG) surgery, type D patients are in worse health than non-type D patients [12].

Mental health treatments, psychological stress reduction, and cardiac rehabilitation may reduce depression in people with CHD, although cardiac rehabilitation is superior in terms of lowering total mortality risk. But mental health treatment is important in cardiac rehabilitation, as evidenced by a growing involvement of mental health practitioners [13].

Post-traumatic stress disorder (PTSD) may play a role in CHD. In PTSD, dysregulation of the hypothalamic-pituitary-adrenal axis, as well as autonomic nerve dysfunction, are widespread, leading to several physiological alterations that can be harmful to the heart. Increased inflammation, vascular endothelial dysfunction, hypercoagulability, and cardiac hyperreactivity have all been observed in PTSD patients. A change in neurochemistry, particularly an increase in arginine vasopressin, as well as a higher incidence of metabolic syndrome, may possibly play a role in poor cardiac outcomes. In older individuals, severe stress may enhance the propensity to hypercoagulability and subsequent hemostasis-related disorders including CHD [14]. Even though the link between PTSD and physical disease is sometimes complicated by health risk behaviors or concurrent psychiatric problems, strong evidence indicates a link between PTSD and CHD [15]. The evidence of a favorable effect of marijuana on PTSD is anecdotal and inconsistent [16].

However, our comparison of the effects of marijuana on red wine is not perfect by any means. Feeling relaxed is an outcome of all the addictions called euphoria; our selection of red wine is based on its anxiolytic effect. Marijuana has been correlated with adverse cardiovascular events, namely tachycardia and possibly MI in people with established heart disease. Red wine has been shown to have beneficial effects on the heart noted above. Therefore, other factors than relaxation may be playing a role in the relationship of marijuana to reduced MI risk we describe here.

A weakness in our study is that it is retrospective and subject to immortal time bias. There is a time interval during which MI cannot occur in the observation period. The participants in the study are “immortal” in the sense that they must live long enough to develop an MI [17].

## Conclusions

Marijuana has been cultivated and used for over 6,000 years, but its cardiovascular and other health impacts have not been thoroughly investigated. The cannabis plant contains more than 100 unique chemical components classified as cannabinoids. One or more of these substances may be responsible for the reduction of MI risk we report here. Further studies are warranted.
